# Cefiderocol pharmacokinetics during acute pulmonary exacerbations in hospitalized adult persons with cystic fibrosis

**DOI:** 10.1128/aac.01539-24

**Published:** 2024-12-10

**Authors:** Christina Koenig, Marguerite L. Monogue, Ryan K. Shields, Colleen M. Sakon, Andrew J. Fratoni, Hanna F. Roenfanz, James D. Finklea, James S. Pope, David P. Nicolau, Joseph L. Kuti

**Affiliations:** 1 Center for Anti-Infective Research & Development, Hartford Hospital 23893 , Hartford, Connecticut, USA; 2 Intensive Care Medicine, University Medical Center Hamburg-Eppendorf 489092 , Hamburg, Germany; 3 The University of Texas Southwestern Medical Center 12334 , Dallas, Texas, USA; 4 University of Pittsburgh Medical Center 6595 , Pittsburgh, Pennsylvania, USA; 5 Indiana University Health 22529 , Indianapolis, Indiana, USA; 6 Division of Pulmonary and Critical Care Medicine, Hartford Hospital 23893 , Hartford, Connecticut, USA; 7 Division of Infectious Diseases, Hartford Hospital 23893 , Hartford, Connecticut, USA; Providence Portland Medical Center, Portland, Oregon, USA

**Keywords:** cystic fibrosis, beta-lactam, cefiderocol, pharmacokinetics

## Abstract

Persons with CF (pwCF) present altered pharmacokinetics (PK) and are often infected with multidrug-resistant (MDR) bacteria. Herein, we describe the PK of cefiderocol, a siderophore cephalosporin with potent activity against MDR Gram-negative rods, in hospitalized adult pwCF with acute pulmonary exacerbation (APE). PwCF received ≥3 doses of 2 g cefiderocol (3 h infusion) with frequency determined according to their estimated glomerular filtration rate (eGFR). Blood sampling collected at steady state. Concentrations were fitted using the non-parametric adaptive grid algorithm in Pmetrics for R. Ten pwCF were enrolled; nine completed the study with six receiving 2 g q8 h and three 2 g q6 h. A two-compartment model best fitted the data. Mean (SD) PK parameters were clearance, 5.66 (1.28) L/h; volume of central compartment, 5.81 (3.52) L, and intercompartment transfer constants, k12, 4.29 (3.46) and k21, 2.25 (2.76) h^−1^. Protein binding was 48% (35–57). The 2 g q8 h regimen achieved a mean free time above the MIC (*f*T >MIC) of 99% (94–99), 90% (69–100), and 64% (41–81) at MICs of 4 (susceptible), 8 (intermediate), and 16 (resistant) mg/L, respectively, with AUC_24h_ of 1,191 (781–1,496) mg/L*h. In pwCF with eGFR >120 mL/min, 2 g q6 h attained 100% *f*T >MIC up to 8 mg/L and 87% (83–92) at 16 mg/L, with AUC_24h_ of 1,279 (1,054–1,590) mg/L*h. Among these nine pwCF with APE with normal or augmented renal clearance, cefiderocol using label prescribed dosing regimens according to eGFR was well tolerated and achieved optimal *f*T >MIC exposure for pathogens up to MICs of 8 mg/L and AUC_24h_ estimates similar to previously reported estimates in non-CF patients.

## INTRODUCTION

Cystic fibrosis is an autosomal, recessive, multi-organ disease caused by variants in the cystic fibrosis transmembrane conductance regulator (CFTR) anion channel (1). CFTR regulates the ion (chloride, bicarbonate) transport and water–electrolyte balance in many organ systems, including the upper and lower airways (2). Thickened secretions and reduced mucociliary transport are consequences of malfunctioning or lacking CFTR channels. Thus, mucus retention, inflammation, airway infection, and acute respiratory symptoms are manifestations of the disease. This provides an optimal environment for microbial colonization by pathogens, including *Pseudomonas aeruginosa*. Despite the implementation of successful early eradication strategies, *P. aeruginosa* contributes to ~50% of infections of acute pulmonary exacerbations (APEs) in adolescent persons with cystic fibrosis (pwCF) (3–5). Therefore, APE usually require intravenous broad-spectrum antibiotics with antipseudomonal activity (6). The presence of multi-drug resistant (MDR) *P. aeruginosa* among pwCF increases with age often leaving few options available to treat APE with MDR organisms (3).

Cefiderocol is a newer siderophore cephalosporin with broad activity against many MDR Gram-negative bacteria that cause APE in pwCF, including *P. aeruginosa, Achromobacter* spp.*,* and *Burkholderia cepacia* complex (7–10). Despite early use for APE due to these challenging pathogens, cefiderocol’s pharmacokinetics in pwCF have not been explored. Notably, it has been hypothesized that malfunctioning CFTR may lead to decreased chloride reabsorption in the tubular lumen and subsequent stimulation of renal secretion (11, 12). Thus, enhanced renal stimulation could lead to increases in total body clearance of various renally cleared drugs, such as previously reported for several beta-lactam antibiotics (13–16). Historically, pwCF often present with low body weight and impaired nutrition status; both factors increasing the risk for hypoalbuminemia, which in turn can lead to altered protein binding affecting efficacy and elimination of drugs (17–20). Herein, we describe the population pharmacokinetics of cefiderocol in hospitalized pwCF presenting with APE.

## RESULTS

### Participants

Ten pwCF were enrolled in the study. One female participant was excluded due to a positive pregnancy test prior to receiving any study drug. Nine pwCF completed the study. Cefiderocol dosing was based on estimated glomerular filtration rates (eGFRs) estimated by Cockcroft–Gault (CG), resulting in three patients who were treated with 2 g q6 h and six patients treated with 2 g q8 h as a prolonged infusion (3 h). Detailed participant data are provided in Table 1. Cefiderocol was well tolerated with no serious adverse events observed during the study duration. One participant experienced mild nausea during the infusion.

**TABLE 1:** Individual demographics and characteristics of the nine persons with cystic fibrosis and acute pulmonary exacerbation enrolled in the study^*a*^

Characteristic	Subject ID									Mean ± SD
	1	2	3	4	5	6	7	8	9	
Age (years)	58	22	34	28	27	46	27	30	28	33 ± 11
Sex (M/F)	M	F	M	M	M	M	M	M	M	NA
Weight (kg)	72	50	63	65	45	78	58	60	64	62 ± 10
Height (cm)	166	157	168	180	168	165	178	177	173	170 ± 7
eGFR (mL/min)	71	99	117	133	118	115	110	123	164	117 ± 24
CFTR modulator use	Yes	Yes	Yes	Yes	Yes	No	Yes	No	No	NA

^*a*^ CFTR, cystic fibrosis transmembrane conductance regulator; eGFR, estimated glomerular filtration rate calculated by Cockcroft–Gault; F, female; M, male; NA, not applicable.

### Population pharmacokinetic analyses

**Fig 1:**
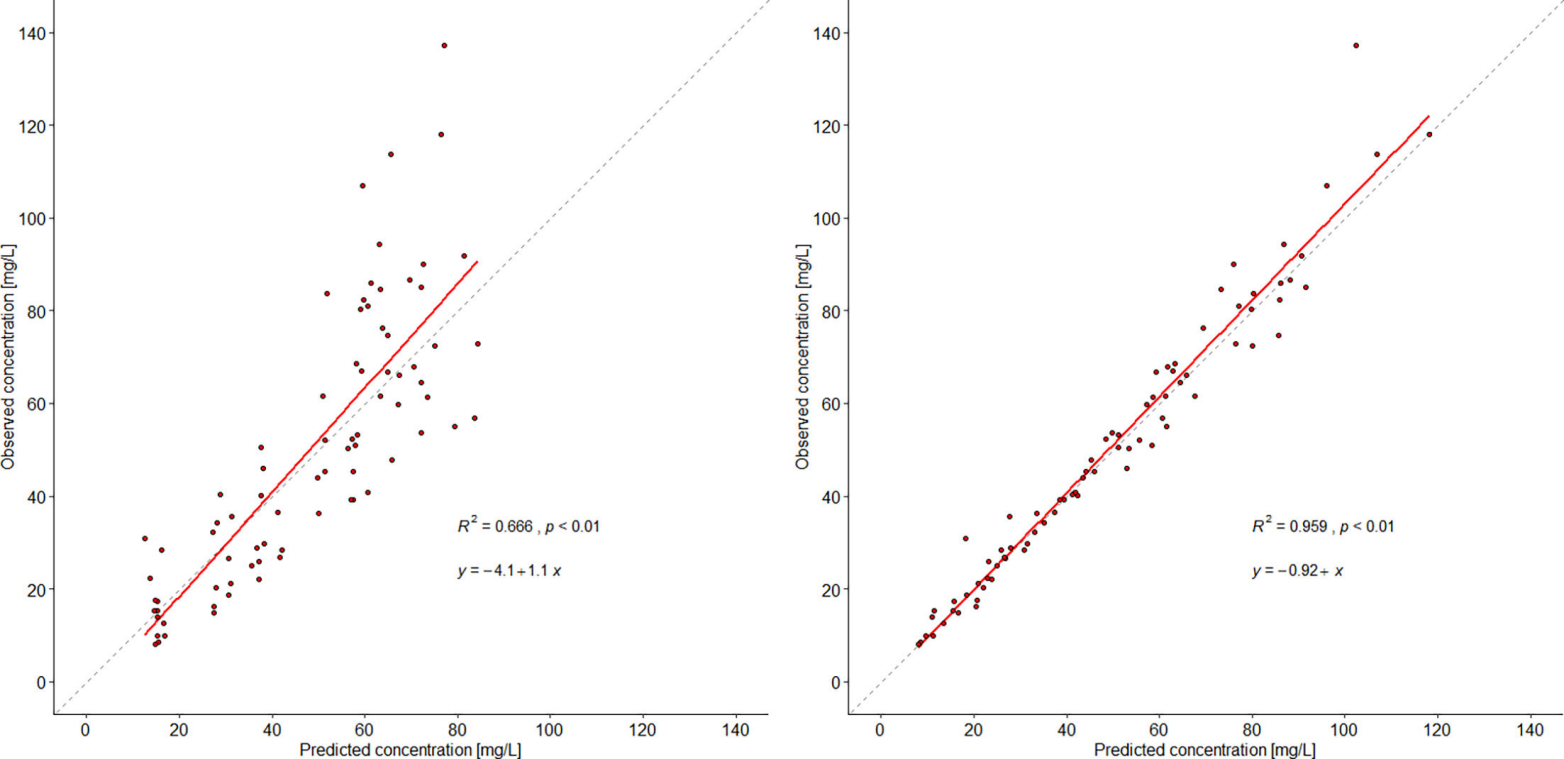
Observed vs (left) population-predicted and (right) maximum *a posteriori* Bayesian individual-predicted cefiderocol concentrations.

A total of 80 blood samples were collected and analyzed for cefiderocol plasma concentrations. Population pharmacokinetic analyses revealed a two-compartment model (Akaike Information Criterion [AIC]: 501) fitted the data better than a one-compartment model (AIC: 548). A linear regression analysis of possible covariates explaining residual variance of total body clearance (CL) and central volume of distribution (V_c_) revealed that eGFR significantly (*P* < 0.05) correlated to CL (Fig. S1). Body weight (BW) was not a significant factor for V_c_ (*P* = 0.76) (Fig. S2). Therefore, eGFR was further investigated in two-compartment models by allometric scaling and utilizing a slope approach (Table S1). However, none of the tested models were superior to the base two-compartment model as indicated by no significant reduction in AIC (difference in AIC between the tested models <2). Therefore, the base two-compartment model was selected as the final pharmacokinetic model. Visual inspections of the goodness-of-fit plots of the observed versus population and Bayesian individual-predicted concentrations showed appropriate spread above and below the line of identity and high R^2^ of 0.96 with a Gamma error of 1.8 (Fig. 1). The final population pharmacokinetic and maximum *a posteriori* (MAP) Bayesian individual parameter estimates are listed in Table 2.

**TABLE 2:** MAP Bayesian and individual pharmacokinetic parameter estimates of the final two-compartment model^*b*^

PK parameter	Population estimate (mean ± SD)	Shrinkage [%]	Individual pharmacokinetic parameter estimates by subject ID								
			1	2	3	4^*a*^	5	6	7	8^*a*^	9^*a*^
CL (L/h)	5.66 ± 1.28	0.08	4.17	4.01	5.20	6.70	5.13	7.68	5.37	5.03	7.59
V_c_ (L)	5.81 ± 3.52	0.12	9.09	5.60	13.21	7.93	6.05	2.88	2.25	2.41	2.87
K_12_ (h^−1^)	4.29 ± 3.46	0.62	0.64	7.11	0.58	1.63	0.50	8.96	4.72	4.82	9.60
K_21_ (h^−1^)	2.25 ± 2.76	0.03	0.92	9.95	1.06	1.77	0.50	1.74	1.15	1.30	1.85

^*a*^ Patients with augmented renal function (eGFR > 120 mL/min).

^*b*^ CL, total body clearance of cefiderocol; V_c_, volume of the central compartment; K_12/21_, intercompartmental transfer constants; MAP, maximum *a posteriori*.

### Individual free time above the minimum inhibitory concentration (%fT >MIC) and total area under the curve (AUC_24h_) exposures

**Fig 2:**
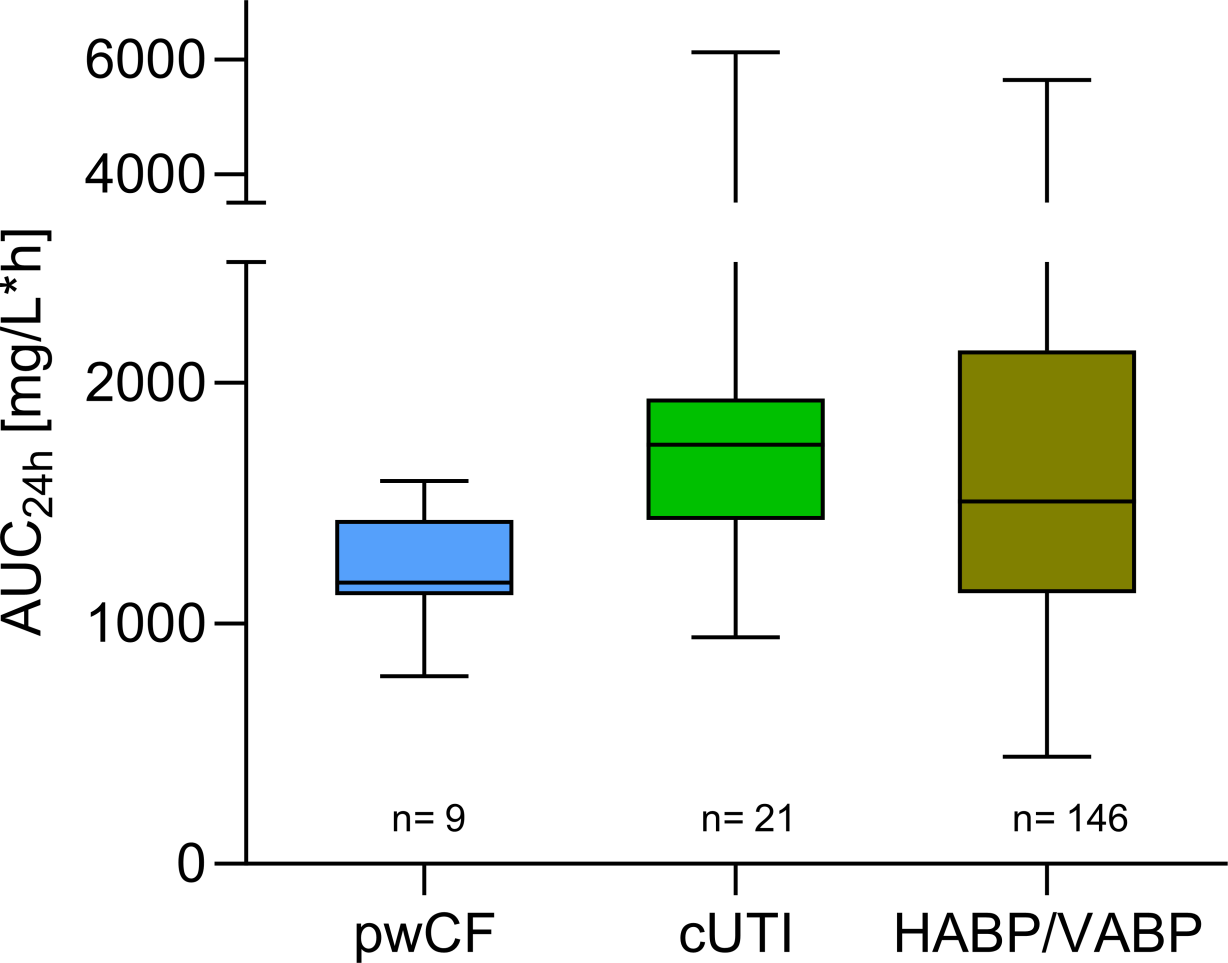
Steady-state total area under concentration time curve (AUC_24h_) comparison of pwCF to patients in the phase III clinical trials (21). Data are presented as median with IQR, whiskers represent the minimum and maximum values. pwCF, persons with cystic fibrosis; cUTI, complicated urinary tract infection; HABP, hospital-acquired bacterial pneumonia; VABP, ventilator-associated bacterial pneumonia.

The mean (range) protein binding for cefiderocol was 48% (35-57). Individual %*f*T > MIC for the dose received is provided in Table 3. Participants receiving 2 g q8 h achieved >90%, 69%–100%, and 41%–81% *f*T > MIC at MICS of 4, 8, and 16 mg/L, respectively, with the administered dosage regimen adapted for their underlying renal function. For those participants presenting with eGFR >120 mL/min, the 2 g q6 h dosing regimen resulted in 100%*f*T > MIC at MICs of 4 and 8 mg/L and 83%–92%*f*T >MIC at 16 mg/L. The mean (range) total cefiderocol AUC_24h_ was 1,221 (781–1,590) mg/L*h; exposures were comparable with those observed in persons without CF enrolled in phase III trials (Fig. 2) (21, 22).

**TABLE 3:** Individual participant cefiderocol exposures simulated based on individual Bayesian pharmacokinetic estimates and free fraction^*a*^

Subject ID	Cefiderocol (g/24 h)	MIC [mg/L]					AUC_24h_ (mg/L*h)	Protein binding (%)
		2	4	8	16	32		
		*fT*_> MIC_						
1	2 g q8 h	100%	100%	100%	81%	34%	1,430	54%
2	2 g q8 h	100%	100%	100%	78%	50%	1,496	45%
3	2 g q8 h	100%	100%	100%	69%	16%	1,153	55%
5	2 g q8 h	100%	100%	88%	59%	38%	1,170	47%
6	2 g q8 h	100%	94%	69%	41%	0%	781	57%
7	2 g q8 h	100%	100%	81%	56%	22%	1,117	56%
Mean		100%	99%	90%	64%	27%	1,191	52%
Min		100%	94%	69%	41%	0%	781	45%
Max		100%	100%	100%	81%	50%	1,496	57%
4	2 g q6 h	100%	100%	100%	88%	46%	1,194	38%
8	2 g q6 h	100%	100%	100%	92%	54%	1,590	49%
9	2 g q6 h	100%	100%	100%	83%	42%	1,054	35%
Mean		100%	100%	100%	87%	47%	1,279	41%
Min		100%	100%	100%	83%	42%	1,054	35%
Max		100%	100%	100%	92%	54%	1,590	49%
Overall results								
Mean		100%	99%	93%	72%	33%	1,221	48%
Min		100%	94%	69%	41%	0%	781	35%
Max		100%	100%	100%	92%	54%	1,590	57%

^*a*^ AUC, area under the concentration time curve.

## DISCUSSION

Cefiderocol is an important addition to the antimicrobial armamentarium against MDR Gram-negative bacteria causing APE in pwCF (3). As a beta-lactam antibiotic, it shows time-dependent bacterial killing with 1 log reduction in CFU achieved at ≥75% *f*T > MIC in a murine thigh infection model (23) and a lung penetration ratio of 34% in non-CF patients with pneumonia (24). The pharmacokinetics of cefiderocol has been studied in various cohorts, including healthy volunteers, patients with renal dysfunction as well as those with active infections (21, 25–30). However, to our knowledge, this is the first report on cefiderocol pharmacokinetics in adult pwCF during an APE. Our results revealed that using label prescribed dosing regimens according to eGFR was well tolerated and provided high %*f*T >MIC and AUC_24_ exposures consistent with non-cystic fibrosis (non-CF) patients.

Cefiderocol concentration time profiles were best described by a two-compartment model. Despite its significant correlation to cefiderocol CL, eGFR was not included in the final model as it did not further improve the model fit, even though its elimination being primarily dependent on glomerular filtration (29). However, previous studies have shown better model performance with eGFR as a covariate on CL for two and three-compartment models in various patient cohorts (27, 30–33). In our study cohort (*n* = 9), renal function (eGFR) calculated by Cockcroft–Gault (CG) only ranged from 71 to 164 mL/min, potentially limiting our ability to observe a significant impact of eGFR on CL in the nested model. The low magnitude on eGFR on CL can also be estimated by the slope (0.039) of the linear regression curve (Fig. S1). However, the mean population CL estimate of 5.66 ± 1.28 L/h is similar to other reports who observed values of 4.0–5.6 L/h for models including eGFR (21, 27, 30). There are observations for other cephalosporins (e.g., ceftolozane and ceftazidime) that show comparable physicochemical and kinetic properties with cefiderocol. Monogue and colleagues found ceftolozane CL in pwCF to also be in line with those of non-CF patients (pwCF CL, 4.76 L/h; non-CF CL, 5.11 L/h) (10). For ceftazidime, CL in pwCF were variable, slightly elevated, and correlated with eGFR but still congruent to CL in healthy volunteers (13, 34, 35). Thus, these changes in elimination were not large enough to conclude general dose adaptions for pwCF are needed. As expected, the estimated CL for the three patients with augmented renal function was numerically higher and ranged from 5 to 7.6 L/h (Table 2). There are hypotheses from the pre-CFTR modulator era regarding how CFTR expression in the kidneys may enhance renal clearance in individuals with cystic fibrosis (pwCF) (2, 11). Notably, with the increased use of CFTR modulators (six of the nine participants were on CFTR modulator therapies during this study), these renal effects may be attenuated in today’s CF patients.

In contrast to other studies, we were not able to identify a significant relationship of actual BW on either CL or V_c_ (18, 34, 35). The small range of BW (45–78 kg) might have attributed to the lack of identifying any correlation if one were to exist. Here, the two-compartment model estimated the V_c_ to be 5.81 ± 3.52 L, which is somewhat lower than previously published data of healthy volunteers (V_c_ = 7.6 L), patients with complicated urinary tract infections (cUTIs) (V_c_ = 7.9 L) and those with hospital-acquired/ventilator-associated bacterial pneumonia (HABP/VABP) and/or sepsis (V_c_ = 7.8 L) (21, 27, 30). However, correction by the mean BW reveals relative V_c_ of 0.09 L/kg BW for these nine pwCF, which resembles the V_c_ (0.11 L/kg BW) of the aforementioned patients.

Mean cefiderocol protein binding among these nine pwCF was 48% (range: 35–57), which is in line with the US-FDA-labeled package insert (Table 3) (22). In contrast to older data, which showed up to twofold higher unbound fractions for some beta-lactam antibiotics in pwCF, we could not identify that magnitude in difference to the available reference data from healthy volunteers (58%) (20, 29). This once again may be related to the introduction of CFTR modulators in the last decade, which were prescribed in the majority of the study participants. These therapies lead to improved general conditions in pwCF and decreased occurrence of malnutrition, which is linked to reduced plasma protein synthesis and hypoalbuminemia (36–39).

Overall, cefiderocol regimens dosed according to eGFR provided >90% *f*T > MIC for susceptible isolates with MICs up to 4 mg/L, exceeding its desired pharmacodynamic target of ≥75% *f*T > MIC derived from murine models (23, 40). Even at a MIC of 8 (intermediate) mg/L, the currently recommended dosing regimens achieved 69%–100% *f*T > MIC dependent on the given regimen (Table 3). Currently reported MIC_50/90_ for cefiderocol against *P. aeruginosa, Achromobacter* spp., and *Burkholderia cepacia* complex are 0.12/0.25, 0.06/0.5, and ≤0.03/0.5 mg/L (41, 42). As a result of the low MIC distribution, cefiderocol administered according to eGFR should provide a high probability of target attainment against this specific population of bacteria often present in pwCF.

The observed mean exposures (AUC_24h_) were 1,191 and 1,279 mg/L*h for patients treated with 2 g q8 h and 2 g q6 h, respectively, which were lower than those achieved in patients from phase III trials (cUTI: 1,944 ± 1,097; HABP/VABP: 1,773 ± 1,503 mg/L). This is likely explained by the targeted smaller range of eGFR in our patient cohort (71–164 mL/min) compared with the wider range (eGFR: 7–540 mL/min) in the phase III trials, which included patients with renal dysfunction (21). It is important to evaluate overall AUC exposure in addition to pharmacodynamic exposure (i.e., *f*T >MIC) so as to avoid higher doses than necessary and minimize the potential for dose related toxicities.

Our study was limited by small numbers (*n* = 9) with a relatively narrow body weight and eGFR ranges, which are common in contemporary pwCF populations. Nonetheless, caution should be taken in extrapolating these findings to pwCF who have higher body weights or might require lower cefiderocol doses due to reduced eGFR. Moreover, we only focused on investigating cefiderocol concentrations in plasma, and thus, exposure into lung tissue may differ from what has been observed in non-cystic fibrosis patients (34% penetration) (24). Only adult pwCF were enrolled in this study, so further studies to characterize cefiderocol pharmacokinetics in pediatric pwCF are still warranted. Finally, this study was not designed to assess clinical or microbiological outcome data.

These are the first data to describe cefiderocol pharmacokinetics, tolerability, and safety in pwCF hospitalized with APE. Using currently labeled dosing recommendations based on estimated eGFR, cefiderocol was well tolerated and achieved >90% *f*T >MIC against susceptible bacteria with MICs up to 4 mg/L in these nine pwCF.

## MATERIALS AND METHODS

### Study design and participants

This was a prospective, multicenter pharmacokinetic study conducted at four sites throughout the US. Patients were eligible if they were >18 years old and experiencing a CF APE. APE was defined as an exacerbation of respiratory symptoms requiring intravenous antibiotics for any four of a catalog of symptoms (see the supplemental material). A documented diagnosis of CF based on the medical history of clinical features of CF, including a positive sweat chloride test or genotypic characterization, was needed for study inclusion. All patients were treated with standard-of-care antibiotics at the discretion of the attending provider. Cefiderocol was administered on top of standard-of-care antibiotics if not already prescribed for treatment. Major exclusion criteria were pregnancy or breastfeeding, hypersensitivity or allergy to any β-lactam antibiotic, moderate to severe renal dysfunction defined as eGFR <60 mL/min based on the Cockcroft–Gault equation. Furthermore, patients with need for any type of renal replacement therapy as well as a history of lung transplant within the last 6 months were not eligible. A safety assessment including clinical and laboratory data was performed during screening, prior, and during study drug infusion as well as at the end of the study (supplemental material).

### Antibiotic administration

Cefiderocol dose and frequency were prescribed according to current approved prescribing information based on eGFR (supplemental material) on the first day of the study drug administration (22). All doses were administered as 3 h prolonged infusions.

### Blood sampling

All participants received at least three doses of cefiderocol to ensure steady-state conditions. Blood was collected in 4 mL K_2_-EDTA vacutainers at 0 (just prior), 1.5, 3, 3.25, 3.5, 4, 5, 6, and 8 h after the start of the final dose. An additional sample (8 mL K_2_EDTA) was collected at 3 h for the determination of cefiderocol protein binding. All samples were stored on ice during transport and centrifuged (1,500×*g*, 10 min, 2°C–8°C) within 30 min of collection. To determine cefiderocol protein binding, an ultrafiltration device (Centrifree, Merck Millipore Ltd., Ireland) and centrifugation (1,500×*g*, 45 min, 2–8°C) was utilized to separate protein-free filtrate (PFF) from plasma in triplicate. All samples were stored at −80°C until quantitative analysis.

### Cefiderocol concentration determination

Cefiderocol plasma (C_Plasma_) and PFF concentrations (C_PFF_) were assayed by a validated ultra-high-performance liquid chromatography with tandem mass spectrometry (LC/MS-MS) (supplemental material). Intra- and interday imprecision was <10% for plasma and PFF quality control samples. The lower limit of quantification was 0.1 mg/L for plasma and 0.05 mg/L for PFF.

### Pharmacokinetic analyses

Cefiderocol concentrations were fitted to one and two-compartment models using the non-parametric adaptive grid algorithm in Pmetrics (Laboratory of Applied Pharmacokinetics, Los Angeles, CA, USA; version 2.1.1) for R (version 4.3.2). The AIC and visual inspection of the goodness-of-fit of the population and individual observed versus predicted plots guided the base model choice. Thereafter, pathophysiologic plausible covariates, such as body size descriptors or calculated eGFR, were analyzed for correlation with pharmacokinetic parameters (i.e., CL and V_c_) using linear regression (Microsoft Excel, 2019; Microsoft Corporation) and evaluated based on R^2^ and *P*-values (*P* < 0.05). For model inclusion, covariate models needed to show a drop of AIC (> 2) compared with the base model and superior model performance was assessed by their goodness-of-fit plots. A gamma error as a scalar on standard deviation (SD) was used in a multiplicative model (C_0_ + C_1_*[obs] + C_2_*[obs]^2^ + C_3_*[obs]^3^) to capture process noise. Gamma model coefficients (Cs) were derived from the inter-run variation coefficients of the cefiderocol assay and were as follows: C_0_ = 0.0068, C_1_ = 0.0585, C_2_ = 0, and C_3_ = 0.

### Individual target attainment analysis

The triplicate PFF concentrations were averaged, and protein binding calculated as follows: protein binding [%] =1- C_PFF_/C_Plasma_ *100. The individual MAP-Bayesian PK parameter estimates and protein binding for each patient were used to simulate %*f*T >MIC exposure for the cefiderocol dose administered during the study.

Within the simulator provided in Pmetrics for R, concentration time profiles (one data point every 15 min) were generated for four doses to achieve steady state. The %*f*T > MIC was determined at MICs of 4, (i.e., the Clinical Laboratory Standards Institute [CLSI] susceptibility breakpoint for *P. aeruginosa*), 8 (intermediate), and 16 mg/L (resistant) (43). To evaluate cefiderocol overall exposures in relation to non-CF patients, total AUC_24h_ was calculated by the linear, log-trapezoidal rule (21, 22).

## SUPPLEMENTAL MATERIAL

Supplemental material aac.01539-24-s0001.docx Study details, LC/MS-MS cefiderocol analytics, and population pharmacokinetic model development.

ASM does not own the copyrights to Supplemental Material that may be linked to, or accessed through, an article. The authors have granted ASM a non-exclusive, world-wide license to publish the Supplemental Material files. Please contact the corresponding author directly for reuse.
