# Supplementary material for: Cefiderocol pharmacokinetics during acute pulmonary exacerbations in hospitalized adult persons with cystic fibrosis
Source: Antimicrob Agents Chemother. 2024 Dec 10;69(1):e01539-24. doi: 10.1128/aac.01539-24 (PMC11784235; doi:10.1128/aac.01539-24)

Version: September 23, 2024

For Submission *Antimicrobial Agents and Chemotherapy*

**Supplemental Material**

**Cefiderocol Pharmacokinetics during Acute Pulmonary Exacerbations in Adult Patients with Cystic Fibrosis**

Christina König^1,2^, Marguerite L. Monogue^3^, Ryan K. Shields^4^, Colleen M. Sakon^5^, Andrew J. Fratoni^1^, Hanna Roenfanz^1^, James D. Finklea^3^, J. Samuel Pope^6^, David P. Nicolau^1,7^, Joseph L. Kuti^1^

**Contents**

[1. APE Criteria catalogue 2](#_Toc176365632)

[2. Exclusion Criteria 2](#_Toc176365633)

[3. Calculation of CrCL using Cockcroft-Gault: 2](#_Toc176365634)

[4. Safety assessment 2](#_Toc176365635)

[a) Baseline clinical and laboratory assessment 2](#_Toc176365636)

[b) Study Day 1 (before cefiderocol infusion) 3](#_Toc176365637)

[c) Study Day 2 3](#_Toc176365638)

[d) End of study visit 3](#_Toc176365639)

[5. Cefiderocol LC/MS-MS method 3](#_Toc176365640)

[6. Covariate Analysis 4](#_Toc176365641)

[7. Model development process 5](#_Toc176365642)

[8. Covariance Matrix of final pharmacokinetic model 6](#_Toc176365643)

[9. Individual predicted vs. observed concentration profiles 7](#_Toc176365644)

# **APE Criteria catalogue**

At least 4 of the following criteria were required to diagnose CF APE: change in sputum; new or increased hemoptysis; increased cough; increased dyspnea; malaise, fatigue, or lethargy; temperature above 38^○^C; anorexia or weight loss; change in physical examination of the chest; decrease in pulmonary function by 10 percent or more from a previously recorded value; or radiographic changes indicative of pulmonary infection.

# **Exclusion Criteria**

- Females that are pregnant and/or breastfeeding
- History of any moderate or severe hypersensitivity or allergic reaction to any β-lactam
- antibiotic (a history of mild rash to a cephalosporin followed by uneventful re-exposure is not a contraindication)
- History of a lung transplant at any time in the past or any other organ transplantation (e.g., liver) within the last 6 months
- Moderate to severe renal dysfunction defined as a CLCR < 60 mL/min (as calculated by the Cockcroft-Gault equation using actual body weight) or requirement for continuous renal replacement therapy or hemodialysis
- History of epilepsy or seizures due to any cause
- Ongoing CF-related liver dysfunction as defined by presence of portal hypertension or cirrhosis
- A hemoglobin less than 8 gm/dL at baseline
- Any rapidly-progressing disease or immediately life-threatening illness (defined as imminent death within 48 hours in the opinion of the investigator)

# **Calculation of CrCL using Cockcroft-Gault:**

Calculated CrCL = (140 - age in years) × ideal body weight (kg)^a,b,c^ / 72 × SCr (mg/dL)

Multiply by 0.85 for female patients

^a^ Ideal body weight (IBW, kg): Males = 50 + [2.3 × (Height (in) - 60)]; Females = 45.5 + [2.3 × (Height (in) - 60)]

^b^ For patients with total body weight (TBW) that is greater than 20% over IBW, use adjusted body weight (ABW, kg): IBW + 0.4*(TBW-IBW)

^c^ If TBW is less than IBW, use TBW in equation

# **Safety assessment**

## **Baseline clinical and laboratory assessment**

*Clinical Assessments:* complete medical and surgical history; performance of a complete physical examination; including respiratory parameters, need for supplemental oxygen (i.e., FiO2) or ventilation; and highest (or lowest if hypothermia) daily temperature (oral, rectal, tympanic, or core) measured; record results of pulmonary function tests available within 1 month of admission for current infection; perform recording of all prior medications taken or received within 3 days before study drug infusion.

*Laboratory:* serum creatinine, blood urea nitrogen, glucose, sodium, potassium, chloride, bicarbonate, magnesium, albumin, total protein, complete blood count (CBC) with differential, total bilirubin, direct bilirubin, alkaline phosphate, alanine aminotransferase, aspartame aminotransferase, and urine analysis with microscopy. A serum hCG test was collected for females of child-bearing potential.

## **Study Day 1 (before cefiderocol infusion)**

- Vital signs (temperature, heart rate, blood pressure, respiratory rate) were recorded within 15 minutes prior to starting the study drug infusion.
- Identification, assessment, and recording of any new adverse events or Serious Adverse Events (SAE).
- Recording of concomitant medications.

## **Study Day 2**

- Concomitant medications were recorded
- Identification, assessment, and recording of any new adverse events or Serious Adverse Events (SAE).

## **End of study visit**

*Clinical Assessments:* Performance of a complete physical examination; examinations conducted as per standard of care were permitted if complete; recordings of resting pulse, blood pressure, including respiratory parameters, need for supplemental oxygen (i.e., FiO2) or ventilation; highest (or lowest if hypothermia) daily temperature (oral, rectal, tympanic, or core) measured; Identification, assessment, and recording of any new adverse events or Serious Adverse Events (SAE).

*Laboratory Assessments*

Serum creatinine, blood urea nitrogen, glucose, sodium, potassium, chloride, bicarbonate, magnesium, albumin, total protein, complete blood count (CBC) with differential, total bilirubin, direct bilirubin, alkaline phosphate, alanine aminotransferase, aspartame aminotransferase, and urine analysis with microscopy.

# **Cefiderocol LC/MS-MS method**

Cefiderocol concentrations were determined using a Waters Acquity UPLC H-Class system with tandem TQ-XS mass spectrometer (LC-MS/MS) equipped with an Acquity UPLC BEH C18, 1.7 μm, 2.1 x 50 mm column maintained at 40 °C. Concentrations of cefiderocol in protein free filtrate (PFF) and human plasma were determined with validated UPLC methods in saline and K_2_EDTA human plasma using cefiderocol-*d*_8_ as the internal standard. PFF samples were analyzed based on the concentration range for the saline method (0.05 to 100 μg/mL). The concentration range for the K_2_EDTA human plasma was 0.1 to 100 μg/mL. Mean interday coefficients of variance (CV) for low and high values of cefiderocol in saline were 4.0% and 5.6%, respectively. Mean interday CV for low and high values of cefiderocol in K_2_EDTA human plasma were 7.7% and 6.4%, respectively. Intraday imprecision was 5.6 and 8.3% for saline and plasma matrices, respectively.

# **Covariate Analysis**

**Figure S1:** CrCL vs. cefiderocol total body CL

CrCl: Creatinine Clearance, CL: Total body clearance

|  |  |  |  |  |  |  |  |
| --- | --- | --- | --- | --- | --- | --- | --- |
|  | *Coefficients* | *Standard Error* | *t Stat* | *P-value* | *Lower 95%* | *Upper 95%* | |
| Intercept | 0.9974 | 1.669 | 0.598 | 0.569 | -2.949 | 4.944 | |
| **CRCL** | 0.0399 | 0.014 | 2.847 | **0.0248** | 0.007 | 0.073 | |

**Figure S2:** Bodyweight vs. cefiderocol central volume of distribution

BW: Bodyweight, V_c_: Central volume of distribution

|  | *Coefficients* | *Standard Error* | *t Stat* | *P-value* | *Lower 95%* | *Upper 95%* |
| --- | --- | --- | --- | --- | --- | --- |
| Intercept | 3.1682 | 8.559 | 0.370 | 0.722 | -17.070 | 23.406 |
| **BW** | 0.0427 | 0.137 | 0.312 | 0.764 | -0.281 | 0.366 |

# **Model development process**

**Table S1:** Performance of tested models

| **Compartments** | **Covariate function on CL** | **AIC** |
| --- | --- | --- |
| 1 | - | 548 |
| 2 | - | 501 |
| 2 | CL=CLi + CLs *CrCL | 509 |
| 2 | CL=CL_0_*(CrCL/117) | 501 |
| 2 | CL=CL_0_*((CrCL/117)^0.75^) | 501 |

AIC: Akaike Information Criterion; CL: Typical body clearance; CLi: Clearance intercept constant; CLs: Clearance slope constant, CL_0_= typical value of clearance for population; CrCL: Creatinine Clearance

# **Covariance Matrix of final pharmacokinetic model**

**Table S2:** Covariance matrix in lower triangular form for the final 2 compartment pharmacokinetic model

|  | **CL** | **V_c_** | **k_12_** | **k_21_** |
| --- | --- | --- | --- | --- |
| **CL** | 1.647 |  |  |  |
| **V_c_** | -1.586 | 12.409 |  |  |
| **k_12_** | 2.322 | -8.819 | 11.966 |  |
| **k_21_** | -1.184 | -0.704 | 3.721 | 7.592 |

CL: Total body clearance, k_12/21_: Intercompartment transfers constants, V_c_: Central volume of distribution

# **Individual predicted vs. observed concentration profiles**

**Figure S3:** Individual predicted vs. observed concentration time profiles for the final population pharmacokinetic model


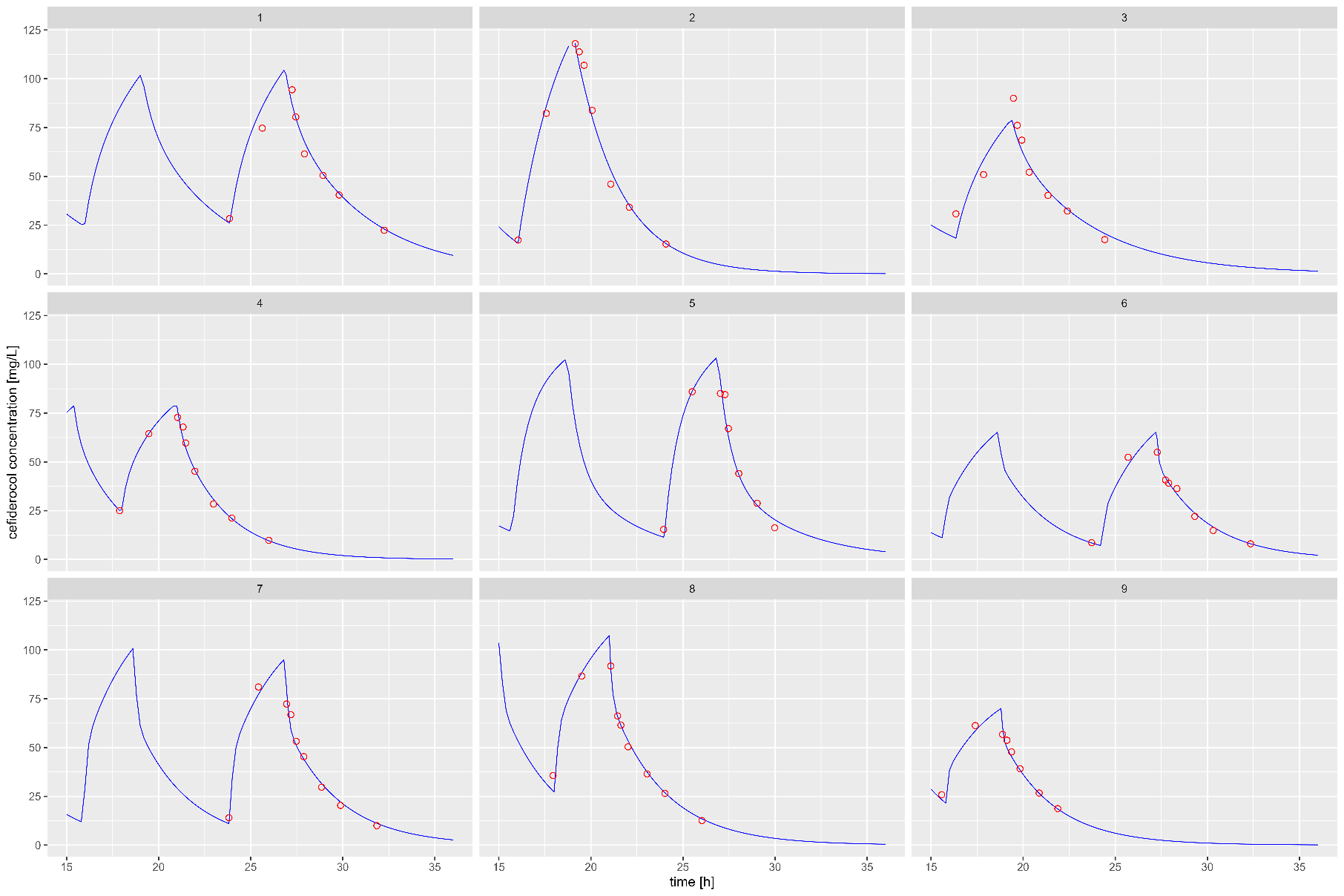

Supplement: Supplemental material — Study details, LC/MS-MS cefiderocol analytics, and population pharmacokinetic model development. [file aac.01539-24-s0001.docx]
